# Development of a conceptual framework for reporting modifications in surgical innovation: scoping review

**DOI:** 10.1093/bjsopen/zrad020

**Published:** 2023-04-27

**Authors:** Sina Hossaini, Christin Hoffmann, Sian Cousins, Natalie Blencowe, Angus G K McNair, Jane M Blazeby, Kerry N L Avery, Shelley Potter, Rhiannon Macefield

**Affiliations:** Department of Population Health Sciences, National Institute for Health and Care Research Bristol Biomedical Research Centre, Bristol Centre for Surgical Research, Bristol Medical School, University of Bristol, Bristol, UK; Department of Population Health Sciences, National Institute for Health and Care Research Bristol Biomedical Research Centre, Bristol Centre for Surgical Research, Bristol Medical School, University of Bristol, Bristol, UK; Department of Population Health Sciences, National Institute for Health and Care Research Bristol Biomedical Research Centre, Bristol Centre for Surgical Research, Bristol Medical School, University of Bristol, Bristol, UK; Department of Population Health Sciences, National Institute for Health and Care Research Bristol Biomedical Research Centre, Bristol Centre for Surgical Research, Bristol Medical School, University of Bristol, Bristol, UK; Department of Gastrointestinal Surgery, North Bristol NHS Trust, Bristol, UK; Department of Population Health Sciences, National Institute for Health and Care Research Bristol Biomedical Research Centre, Bristol Centre for Surgical Research, Bristol Medical School, University of Bristol, Bristol, UK; Department of Gastrointestinal Surgery, North Bristol NHS Trust, Bristol, UK; Department of Population Health Sciences, National Institute for Health and Care Research Bristol Biomedical Research Centre, Bristol Centre for Surgical Research, Bristol Medical School, University of Bristol, Bristol, UK; Division of Surgery, Bristol Royal Infirmary, University Hospitals Bristol and Weston NHS Foundation Trust, Bristol, UK; Department of Population Health Sciences, National Institute for Health and Care Research Bristol Biomedical Research Centre, Bristol Centre for Surgical Research, Bristol Medical School, University of Bristol, Bristol, UK; Department of Population Health Sciences, National Institute for Health and Care Research Bristol Biomedical Research Centre, Bristol Centre for Surgical Research, Bristol Medical School, University of Bristol, Bristol, UK; Bristol Breast Care Centre, North Bristol NHS Trust, Bristol, UK; Department of Population Health Sciences, National Institute for Health and Care Research Bristol Biomedical Research Centre, Bristol Centre for Surgical Research, Bristol Medical School, University of Bristol, Bristol, UK

## Abstract

**Background:**

Innovative surgical procedures and devices are often modified throughout their development and introduction into clinical practice. A systematic approach to reporting modifications may support shared learning and foster safe and transparent innovation. Definitions of ‘modifications’, and how they are conceptualized and classified so they can be reported and shared effectively, however, are lacking. This study aimed to explore and summarize existing definitions, perceptions, classifications and views on modification reporting to develop a conceptual framework for understanding and reporting modifications.

**Methods:**

A scoping review was conducted in accordance with PRISMA Extension for Scoping Reviews (PRISMA-ScR) guidelines. Targeted searches and two database searches were performed to identify relevant opinion pieces and review articles. Included were articles relating to modifications to surgical procedures/devices. Data regarding definitions, perceptions and classifications of modifications, and views on modification reporting were extracted verbatim. Thematic analysis was undertaken to identify themes, which informed development of the conceptual framework.

**Results:**

Forty-nine articles were included. Eight articles included systems for classifying modifications, but no articles reported an explicit definition of modifications. Some 13 themes relating to perception of modifications were identified. The derived conceptual framework comprises three overarching components: baseline data about modifications, details about modifications and impact/consequences of modifications.

**Conclusion:**

A conceptual framework for understanding and reporting modifications that occur during surgical innovation has been developed. This is a first necessary step to support consistent and transparent reporting of modifications, to facilitate shared learning and incremental innovation of surgical procedures/devices. Testing and operationalization is now needed to realize the value of this framework.

## Teaser

This paper describes the conduct of a scoping review and the development of a conceptual framework for reporting modifications in surgical innovation.

## Introduction

Surgical innovation has the potential to improve outcomes for patients and advance standards of surgical care^1^. Innovation in surgical practice can include new surgical techniques or devices (for example minimally invasive surgery) or can involve incremental changes to existing methods and devices (for example technological improvements)^2,3^. In the early stages of surgical innovation, new techniques or devices are modified or refined as innovations are optimized^4^. Whilst modifications may be made with the intention to improve or optimize innovative procedures or devices, this can be associated with uncertainty, unknown risks, and sometimes adverse events and harm. Understanding and sharing how and why modifications are made is therefore important for safe and efficient innovation.

Frameworks to improve transparency of surgical innovation and facilitate standardized approaches to evaluating surgical innovation have been introduced. The Idea, Development, Exploration, Assessment and Long-Term follow-up (IDEAL) framework, for example, outlines a pathway of five stages in surgical innovation (from first in human to long-term study) with recommendations on how new surgical procedures and devices should be developed and evaluated at each stage^4,5^. A core outcome set for standardized evaluation of innovative surgical procedures and devices (COHESIVE COS) has recently been developed to define outcomes to be measured and reported in early phase studies of surgical innovation^6,7^. Reporting of modifications is a key component of both stages 2a/2b within the IDEAL framework. Reporting of modification is also recommended as part of the recently developed COHESIVE COS, which was identified as a core outcome domain to report all early phase studies of surgical procedures/devices^6^. This included modifications to technical components, patient selection criteria and co-interventions (for example administration of intravenous analgesia). There are currently, however, no standardized frameworks for reporting modifications in studies of surgical innovation. Indeed, vague terminology is sometimes seen in literature/guidance without further definition or explanation, for example referring to modifications as ‘major’ or ‘minor’^8,9^. Current approaches to reporting modifications in surgical innovation, and the incremental learning arising from them, are informal and inconsistent^2^. Deficient reporting and a lack of opportunity for sharing incremental learning may result in surgeons unknowingly repeating ineffective or detrimental modifications and may increase the risk of avoidable patient harm. In contrast, sharing experiences of effective modifications may also be compromised, meaning efficient uptake of promising innovations is hindered. A systematic and transparent approach to reporting modifications when introducing new procedures or devices into clinical practice is recommended to support shared learning, promote efficiency and protect patient safety^10^.

This study aimed to explore and summarize existing literature about modifications related to: definitions, perception, classifications and views on modification reporting, to inform a conceptual framework for understanding and reporting modifications in surgical innovation.

## Methods

A scoping review was conducted and reported in accordance with relevant PRISMA guidelines (PRISMA-ScR, see *Appendix S1*)^11^. The multidisciplinary study team consisted of surgeons, methodologists and trialists with extensive experience in health services research. A study protocol has previously been published^12^. Ethical approval was not required for this review as no empirical patient data or sensitive information was collected.

### Search strategy

Multiple consecutive searches were required to identify relevant articles due to a lack of index terms for the specific subject areas of surgical innovation and modifications^5,13,14^. Targeted internet searches using keywords for ‘modifications’, ‘definition’, ‘classification’ and ‘invasive procedures’ were initially performed to identify relevant key papers. The terms used in electronic medical databases for indexing the key papers were identified, including additional keywords, and were used to inform the search terms for the current review. Two separate database searches were conducted in MEDLINE (Ovid version) to identify review articles and opinion pieces. Snowball searching (that is backward and forward searching of reference lists) was completed for all articles included in the review, to identify further relevant articles^15^. Searches were conducted between July and October 2020. The search strategy is detailed in *Tables S1*, *S2*.

### Study eligibility

Included were any review articles or opinion pieces published in a peer-reviewed journal and relevant to surgical innovation (that is topics related to the development or use of new surgical procedures and devices), that provided one or more of the following: a scientific or descriptive definition of modifications; a description of how modifications may be perceived or understood; a classification, typology and/or taxonomy for categorizing modifications; and views and/or opinions on how modifications could be reported.

Excluded were non-English language publications, conference abstracts and studies reporting empirical examples of modifications. Review articles published before 2001 were excluded to allow for an anticipated manageable number of records to be screened whilst maintaining a broad time interval. Publications within the last 20 years were included because this time frame was identified as an interval of increasing interest in methodology relating to evaluation of surgical innovation^2,16^. No publication date limit was set for opinion pieces because fewer records were retrieved.

### Study selection

A two-stage screening process was performed independently by two reviewers (S.H., C.H.). First, titles, abstracts and keywords were examined to identify potentially eligible articles. This was followed by an in-depth full-text review. All articles where inclusion was uncertain were discussed between the two reviewers and assessed independently by a third reviewer (R.M.) to reach a final consensus on their inclusion. Input from the wider study team was sought wherever necessary.

### Data extraction and analysis

Data extraction was performed independently by two reviewers (C.H., S.H.) using a predesigned standardized proforma, directly into an electronic database (see *Table S3*). Data related to the definition, perception, classification or views on reporting of modifications were extracted verbatim. Publication characteristics, author affiliation, conflict of interest, funding statements and institution characteristics were recorded to examine any patterns in potential influences on the available evidence. Initially, each reviewer independently extracted data from the same five articles. Extracted data were then compared and discussed to ensure that subsequent data extraction was consistent and sufficient quality, that is extracted data met one of the four study eligibility criteria specified above. Data extraction of remaining articles was performed independently by the two reviewers, with regular meetings for further quality assurance.

Article types were grouped into categories (for example commentaries, letters and perspectives were grouped as ‘opinion pieces’). Publication characteristics were summarized using descriptive statistics. Other verbatim extracted data relating to the review objectives were analysed using a narrative synthesis to identify and iteratively derive common themes using principles of thematic analysis, consisting of data familiarization, systematic coding of textual data, and iterative refinement of themes and subthemes^17,18^. Where a narrative synthesis was not possible due to a paucity of data for a specific review objective, content analysis (quantifying the presence of certain words) of extracted data was performed to explore the terminology used in the included articles. Narrative synthesis was performed separately for articles relating to surgical procedures and devices. Analyses were assisted using qualitative analysis software (NVivo; version 12)^19^.

### Development of a conceptual framework

**Fig. 1:**
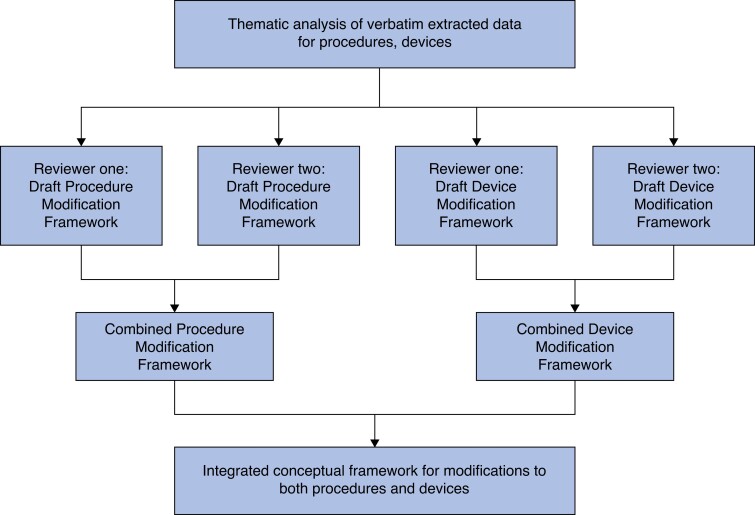
Overview of steps in the development of an integrated conceptual framework for modifications to procedures and devices

A conceptual framework for modifications was developed following the principles of framework analysis in three steps^20,21^. First, the inductively derived themes were reviewed to qualitatively compare their content, with a view to highlighting similarities and differences (for example whilst several articles described the broader concept of underlying drivers for modifications, they differed in the specific justifications described, such as improving patient outcomes, resolving technical problems or expanding patient selection)^22–25^. Second, themes considered to be similar were grouped, and subsequently organized according to relationships between them. Two reviewers (C.H., S.H.) independently developed draft conceptual frameworks informed by these themes, separately for surgical procedures and devices to ensure maximum validity. Third, the reviewers’ independent draft frameworks were compared and discussed between the reviewers (C.H., S.H.) and combined to produce agreed frameworks for surgical procedures and devices. Finally, through further rounds of discussion with the wider study team, the two frameworks were integrated to produce a single conceptual framework for modifications to procedures and devices, focusing on applicability of the framework to clinical practice. This process is summarized in *Fig. 1*. Existing reviews of empirical studies of surgical innovation known to the study team^5,7,13,14^ were examined to aid organization of the framework throughout this process.

## Results

**Fig. 2:**
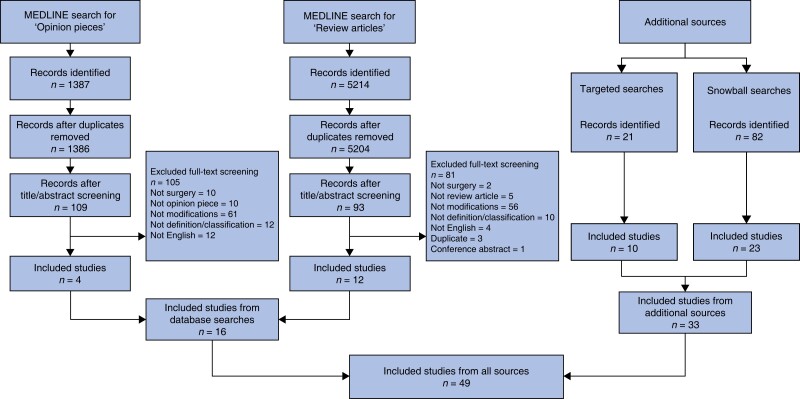
PRISMA flow diagram

The database search yielded a total of 6601 records (*Fig. 2*). Of these, 1387 and 5214 records were identified through searches for opinion pieces and review articles respectively. Additional targeted and snowball searches identified a further 103 potentially relevant articles. A total of 49 articles were eligible for analysis.

### Study characteristics

Characteristics of included articles are summarized in *Table 1* and *Table S4*. Article types were predominately narrative reviews (*N* = 22, 45 per cent) and opinion pieces (*N* = 14, 29 per cent). Most articles were relevant to surgical procedures (*N* = 37, 76 per cent) and discussed procedures generically (*N* = 31, 63 per cent) rather than referring to a specific operative technique (*N* = 6, 12 per cent). Most articles discussed surgical devices in general (*N* = 15, 31 per cent), whilst others discussed a specific device (*N* = 6, 12 per cent). Most articles were published by author(s) affiliated to higher education institutions such as universities and teaching hospitals (*N* = 41, 84 per cent) and conducted in the USA (*N* = 28, 57 per cent), followed by Europe (*N* = 23, 47 per cent). Included articles were mostly published in the last decade (*N* = 30, 32 per cent).

**Table 1:** Summary of characteristics of included articles

	*n* (%)
**Type of article**	
Narrative review	22 (45)
Opinion pieces (comments, debates, editorials)	14 (29)
Guideline	7 (14)
Database review	4 (8)
Systematic review	2 (4)
**General topics discussed**	
Surgical procedures*	37 (76)
Surgical devices*	20 (41)
Both	8 (16)
Details about content of the article	
Surgical procedures in general	31 (63)
Surgical devices in general	15 (31)
Specific surgical technique	6 (12)
Specific surgical specialty	6 (12)
**Conflict of interest**	
Conflict of interest statement present	38 (78)
Conflict of interest statement not present	11 (22)
Details about presented statements	
Authors declared no conflict of interest	27 (55)
Statement is unclear	9 (18)
Authors declared a conflict of interest	2 (4)
**Funding**	
Funding or sponsorship statement present	36 (73)
Funding or sponsorship statement not present	13 (27)
Details about presented statements	
Funding or sponsorship was received	15 (31)
No funding or sponsorship was received	13 (26)
Statement is unclear	8 (16)
**Year of publication**	
2020–2016	15 (31)
2015–2011	15 (31)
2010–2006	12 (24)
2005–1999	7 (14)
**Authors' affiliation**	
Higher education affiliation	41 (84)
Research collaboration	3 (6)
Industry affiliation	2 (4)
Country*	
USA	28 (57)
Europe	23 (47)
Canada	7 (14)
Asia	4 (8)
Single country	41 (84)
Multinational	8 (16)

* Categories are not mutually exclusive.

### Definitions, perception, classifications and views on how to report modifications

#### Definitions

No scientific or descriptive definition of modifications was identified in the included articles.

#### Classifications and views on modification reporting

The included articles referenced a total of eight different classifications (see *Table S5*). The most frequently cited were the IDEAL framework, which proposes reporting of iterative modifications in the early stages of surgical innovation^4,7,26–29^, and the US Food and Drug Administration (FDA) Premarket Approval (PMA) pathway^0–4^, which describes the FDA regulatory framework through which modifications to devices are approved.

#### Perceptions

A total of 13 themes were identified from verbatim extracted descriptions of modifications (see *Table S6*). Of these, six themes were common to both procedures and devices and are described in more detail below. A summary of these six themes with example extracts from the included articles can be found in *Table S7*.

Three further themes were unique to procedures: phase of surgical innovation (that is modifications are a necessary stage in the development of procedures with an aim to reach a point of stability); failure and mistakes (that is are inevitable elements of modifications which should be (prospectively) defined and responded to); increased complexity (that is modifications can lead to technically more complex procedures to achieve better outcomes, which requires managing potentially conflicting outcomes). The remaining four themes were unique to devices which are characterized by: transparency issues (that is lack of comprehensive reporting of information primarily related to minor device modifications, which require improvements in regulation, oversight, registries and/or greater risk management); collaboration with industry (that is the need for joint working between industry, regulatory bodies and clinicians); access (that is the perceived right for patients to access innovation which often justifies rapid introduction of modified devices) and the relationship between surgeon learning and patient selection (that is changes in patient selection criteria in response to the surgeon’s learning curve).

#### Perceptions of modifications common to both procedures and devices

##### Modifications as a process

Modifications were often described as a process in relation to both procedures and devices. Modifications to procedures were characterized as a stepwise and goal-directed process^23,24,35^. Further descriptions revealed goals may be changeable and evolving with experience, fixed (for example clinical outcomes, measures of failure and success) or independent of clinical outcomes (for example practical concerns)^23,24^. In contrast, modifications to devices were viewed as a dynamic process during which changes occur frequently and continuously^36^. This process was described as an open-ended cycle (without an end goal) of postmarket product development, where incremental minor improvements to devices can lead to an entirely new product emerging over time. Reference was made to existing US FDA regulations facilitating this process and labelled ‘predicate creep’ or ‘design drift’^37,38^.

##### Types/understandings of modifications

Articles reported different types/understandings of modifications for both procedures and devices. Changes to technical components of the surgery, physical changes to the device, changes to patient selection and changes to co-interventions were all described. Two distinct types of modification were evident from descriptions of surgical procedures: a change to a single aspect (for example a technical modification) of an existing procedure which may lead to an entirely new procedure or multiple, cumulative changes to different aspects of a procedure (including new indication, combining of established techniques)^39,40^. In contrast, device modifications were broadly described as either: manufacturer-led modifications that were primarily design-focused and clinician-led modifications that focused on addressing a clinical need^31,41^.

##### Magnitude of modifications

Articles referred to the magnitude of modifications for both procedures and devices, broadly described as ‘major’ and ‘minor’. ‘Major’ procedure modifications were considered to represent changes conferring greater levels of risk or uncertainty, necessitating greater surgeon training needs and performance assessment, or requiring external scrutiny^42^. ‘Minor’ procedure modifications were consistent with smaller technical modifications within an accepted procedure, requiring less extensive surgeon training. For surgical devices, articles referred to ‘minor’ device modifications such as smaller design changes, in contrast to ‘major’ device modifications that might include new indications for use^32^.

##### Drivers for modifications

In some articles, modifications were described as being motivated by underlying rationale that could be considered to represent ‘drivers’ for modifications (that is the reasons that influence why modifications are made). Examples included the desire to advance existing treatments/foster further innovation, improve existing techniques, improve outcomes, respond to technical problems/failures, improve efficiency or broaden patient selection/indication^24,43^.

##### Enablers of modifications

Modifications were described in some articles as being influenced by ‘enablers’ (that is factors that allow or make modifications easier to happen) or facilitated the stepwise process of development^43,44^. For example iterative developments may be influenced by the dissemination of existing innovations or technological advancements, or by shared learning.

##### Relationship to formal research

Some articles referred to the limitations of current research or governance structures in identifying the need for oversight of modified procedures. Other articles considered how evolving procedures or devices that were continually being modified, presented researchers with challenges when interpreting short- *versus* long-term safety and effectiveness data from clinical studies^44,45^.

### Conceptual framework for understanding and reporting modifications

**Fig. 3:**
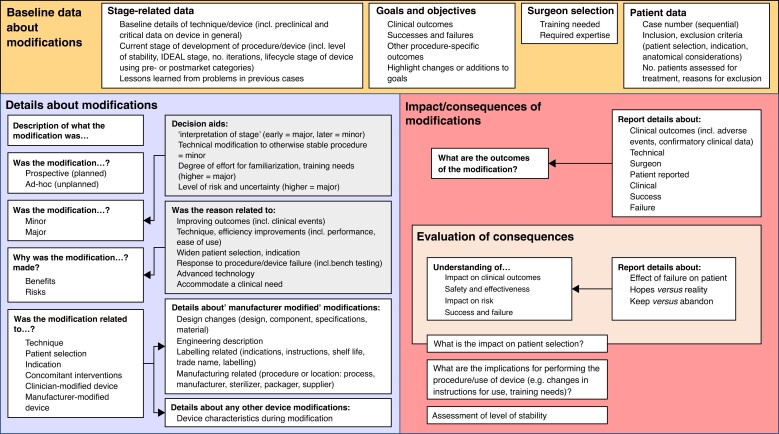
Integrated conceptual framework for modifications to procedures and devices

The themes listed above were organized to produce draft conceptual frameworks for reporting modifications, separately for procedures and devices (*Figs S1*, *S2*). *Figure 3* presents the single integrated conceptual framework relevant to modifications to procedures and devices. The framework comprises three overarching components to represent the organized themes derived from the data: baseline data about modifications, details about modifications and impact/consequences of modifications.

The ‘baseline data about modifications’ component of the integrated conceptual framework broadly includes background contextual features that may be relevant to understanding modifications to a procedure or device. Within this component, the stage of development, goals and objectives, patient data (for example patient selection criteria/indication and reasons for exclusion) and lessons learned from previous cases may contextualize modifications to procedures or devices. The ‘details about modifications’ component broadly outlines the modification itself and includes other identified themes relevant to understanding the modification, for example what aspect of the procedure/device the modification related to, why the modification was made and the perceived magnitude of the modification. The ‘impact/consequences of modifications’ component includes an assessment of actual or perceived outcomes of a modification, and considers the subsequent implications on future cases, including an assessment of whether the modification might be kept, abandoned or modified further.

## Discussion

This study systematically analysed existing literature to identify definitions, perception, classifications, and views on modifications to surgical procedures and devices, with the aim of developing a conceptual framework for understanding and reporting modifications undertaken during early phase studies. Syntheses of verbatim data, extracted from 49 review articles and opinion pieces, identified 13 relevant themes. Six themes were common to modifications to procedures and devices, whilst three themes were unique to procedures and four unique to devices. All themes were organized to develop an integrated conceptual reporting framework for modifications to procedures and devices, comprising three overarching components: baseline data about modifications, details about modifications and impact/consequences of modifications. Use of the framework is recommended to increase transparency in surgical innovation and enhance the safe and efficient introduction of new procedures and devices into clinical practice.

This work completes an initial step towards operationalizing a core outcome domain of the COHESIVE COS, which recommends reporting modifications in all studies of early phase surgical procedures and devices^6^. Prior studies have highlighted considerable heterogeneity when reporting innovation-specific outcomes including modifications^13^. The proposed framework provides a summary of the conceptual areas that may be considered relevant to informing a future reporting framework for modifications. The framework can be used in context alongside existing guidance for reporting surgical innovation. For example the IDEAL checklist, developed to facilitate reporting for surgical studies throughout their lifecycle specifies a minimum list of reporting items, including broad information about modifications in stages 2a and 2b (for example number, time point, magnitude and reason for modifications made)^7^. Findings from the current study complement these recommendations to facilitate how modifications can be conceptualized, described and reported in detail. The framework may, for example help surgeons to determine a modification’s magnitude and describe their rationale for making the modification, ultimately facilitating shared learning. The eight classifications identified in this review may also provide helpful systems in deciding how to adopt processes for recording information in specific settings.

Findings from this review emphasize the important role of modifications during the development phase of innovative surgical devices and procedures. Understanding when and why modifications occur in practice is crucial for shared learning of surgical innovation. Importantly, improved understanding and real-time sharing of modifications may accelerate efficient evaluation of innovative procedures and devices. Modifications can serve as an indicator of the stability of innovative procedures and devices, crucial to establishing the point at which definite evaluation through an RCT is recommended^46^,^47^. It is anticipated that the framework will be useful to those reporting innovation in real-time, to indicate whether a procedure/device has sufficiently stabilized and is ready for evaluation in an RCT. It may also be useful, for example to establish the stage of innovation (that is level of stabilization of a new procedure/device) retrospectively^48^.

There is no agreement in the current literature about how to define modifications, and no definitions were identified in this review. Further work and stakeholder consensus is therefore needed to achieve a commonly accepted definition to progress standardized reporting. However, challenges in this regard have been noted during attempts to define ‘surgical innovation’ which was found to be underpinned by conceptual areas rather than a single, practical definition^44,49^. The conceptual framework proposed in this study may be used to inform work to define modifications in surgical innovation.

This study had several strengths. Robust scoping review methodology was used to examine existing literature on a topic area with little prior exploration^11^. The search strategy was comprehensive and designed to identify potentially relevant literature in a broad range of article types, which were considered to be specifically relevant to achieving the review objectives. A deliberately broad approach was applied during the conduct of this study to include as many potentially relevant articles as possible and to ensure no important evidence would be excluded. This means that there was no a priori definition of a reporting or classification system, however, synthesis included important aspects of modifications that may have been overlooked in other studies and therefore allowed development of a comprehensive conceptual framework. Specifically, the study adopted a broad view of modifications as suggested in the COHESIVE COS (for example modifications to patient selection criteria and co-interventions) and included evidence for modifications to procedures and devices by undertaking multiple searches (two databases, targeted and snowball searches)^6^. The approach to data analysis consisted of established qualitative methods, drawing on both thematic analysis and on principles of framework analysis to inductively generate an understanding of important concepts and organize themes^17,18^. Scoping review methodology has been recognized as a valid and insightful means to synthesize evidence in healthcare and various other areas of research with little prior evidence^50–52^. Framework analyses is an established method for organizing data^20,21^ and has been previously applied to develop conceptual frameworks in healthcare^53^ or reporting frameworks in other fields^54,55^. Synthesis was conducted by two independent experienced reviewers with input from a multidisciplinary team consisting of methodologists, health services researchers and surgeons to ensure rigour.

Study limitations should also be noted. It is acknowledged that some relevant publications may have been missed, as identification of all potentially relevant articles remains dependent on indexing. Included articles were limited to those published in the English language and from peer-reviewed journals from mostly higher education institutions and academic surgeons. This could potentially have limited the ‘real-world’ understanding of modifications in other types of clinical settings or institutions. Likewise, it is unknown whether the conceptual framework is appropriate in wider settings (for example modifications to an entire technique *versus* a small component of an existing procedure). Refinements of the conceptual framework to accommodate such variability may be needed in future work to accelerate standardized reporting of modifications and evaluation of surgical innovation.

This work has completed a necessary first step and developed a conceptual framework to understand modifications to innovative procedures and devices. Testing and operationalizing the proposed conceptual framework into a validated and clinically applicable tool for identifying, describing and reporting modifications are now needed, alongside identification of optimal ways to integrate findings into practice. A validated reporting framework for modifications will complement the Template for Intervention Description and Replication (TIDieR) checklist and CONSORT statement for Non-Pharmacological Treatments (NPT), which both refer to modifications and tailoring but lack detail in several areas relevant to effective reporting^56,57^. This is being explored within the National Institute for Health and Care Research (NIHR) funded Bristol Biomedical Research Centre (BRC) in the UK, which aims to improve the introduction and evaluation of innovative surgical procedures and devices.

Routine use of a framework to describe and report modifications will support a more systematic and standardized approach to reporting modifications and ultimately increase transparency in surgical innovation, facilitate shared learning amongst surgeon innovators and minimize research waste and patient harm.

## Supplementary Material

zrad020_Supplementary_Data

## Data Availability

No new data were collected or generated from this study. All study materials have been made available in the supplementary file.
