# Supplementary material for: Development of a conceptual framework for reporting modifications in surgical innovation: scoping review
Source: BJS Open. 2023 Apr 27;7(2):zrad020. doi: 10.1093/bjsopen/zrad020 (PMC10139440; doi:10.1093/bjsopen/zrad020)
Supplement: zrad020_Supplementary_Data [file zrad020_supplementary_data.docx]

**Development of a conceptual framework for reporting modifications in surgical innovation: scoping review**

**Sina Hossaini*^1 ƚ^ BMBS**, Christin Hoffmann*^1^ PhD, Sian Cousins^1^ PhD, Natalie Blencowe^1,2^ PhD, Angus G K McNair^1,2^ PhD, Jane M Blazeby^1,3^ MD, Kerry N L Avery**^1^ PhD, Shelley Potter**^1,4^ PhD, Rhiannon Macefield**^1^ PhD.

*Joint first author, **Joint senior author

^1^ National Institute for Health and Care Research Bristol Biomedical Research Centre, Bristol Centre for Surgical Research, Population Health Sciences, Bristol Medical School, University of Bristol, 39 Whatley Road, Bristol, BS8 2PS, UK

^2^ Department of Gastrointestinal Surgery, North Bristol NHS Trust, Southmead Road, Bristol, BS10 5NB, UK

^3^ Division of Surgery, Bristol Royal Infirmary, University Hospitals Bristol and Weston NHS Foundation Trust, Bristol, BS2 8HW, UK

^4^ Bristol Breast Care Centre, North Bristol NHS Trust, Southmead Road, Bristol, BS10 5NB, UK

^ƚ^ Corresponding author and requests for reprints: Mr Sina Hossaini, Bristol Centre for Surgical Research: Population Health Sciences, University of Bristol, 39 Whatley Road, Bristol, BS8 2PS, United Kingdom. Telephone: (+44) 01179 287279. Email: [sina.hossaini@bristol.ac.uk](mailto:sina.hossaini@bristol.ac.uk). ORCID ID: <https://orcid.org/0000-0001-8122-5321>.

**Supplementary Materials - Index**

# Table of **contents**

| **Title & Contents** | **Page(s)** |
| --- | --- |
| **SUPPLEMENTARY METHODS** | |
| **Search strategies**   - Table S1: search strategy for review articles - Table S2: search strategy for opinion pieces | 3 – 6 |
| **Data extraction**   - Table S3: summary of data extraction proforma | 7 |
| **SUPPLEMENTARY RESULTS** | |
| **Data Table**   - Table S4: summary of included studies | 8 – 9 |
| **Classification systems, taxonomies, views on reporting**   - Table S5: Summary of classification systems, taxonomies or typologies for categorizing or reporting modifications | 10 – 11 |
| **Themes related to perceptions of modifications**   - Table S6: Summary of themes for perceptions of modifications to surgical procedures and devices | 12 |
| **Summary of common themes**   - Table S7: Common themes for procedures and devices from thematic analysis of data on perceptions of modifications | 13 – 14 |
| **Draft conceptual frameworks**   - Figure S1: Combined procedure modification conceptual framework - Figure S2: Combined device modification conceptual framework | 15 |
| **SUPPLEMENTARY APPENDIXES** | |
| **Preferred Reporting Items for Systematic reviews and Meta-Analyses extension for Scoping Reviews (PRISMA-ScR) Checklist**   - Appendix S1: PRISMA ScR Checklist | 16 – 17 |
| **References** | 18 – 20 |

# **Search strategies**

Detailed methods about the development of the search strategy are reported in a published protocol (1). In brief, a systematic search for review articles and opinion pieces was performed in MEDLINE (Ovid version), using Boolean operators to combine keywords related to themes of “definition, perception, classification”, “invasive procedures” and “modifications”.

***TABLE S1 – Search strategy for review articles***

| **Definition, perception, classification** |
| --- |
| 1. Glossar*.tw. 2. Typolog*.tw. 3. Categori*.tw. 4. Framework?.tw. 5. Taxonom*.tw. 6. Recommendation?.tw. 7. Codif*.tw. 8. Catalogu*.tw. 9. Terminolog*.tw. 10. Concept*.tw. 11. ((defin* or classif* or type?) adj3 (modification? or change?)).tw. 12. (method? adj2 (describ*3 or classif*3 or report*3 or categor*)).tw. 13. or/1-12 |
| **AND** |
| **Modifications** |
| 1. Modification?.tw. 2. (Incremental adj3 innovation?).tw 3. (adjust* or iterat* or alternat* or adapt* or revis* or switch* or custom* or re-work* or tweak* or tinker* or refin* modif* or improv* or combin* or nuance* or evolv* or add* or var* or chang* or alter* or deviat* or develop* or differ* or similar) adj3 (surg* or operat* or intra-operative or peri-operative or procedure* or device?).tw 4. OR 1-3 |
| **AND** |
| **Invasive Procedure** |
| 1. invasive.tw. 2. incision.tw. 3. cut.tw. 4. percutaneous.tw. 5. puncture.tw. 6. (natural adj1 orifice).tw. 7. endoscop*.tw. 8. colonoscop*.tw. 9. gastroscop*.tw. 10. laparoscop*.tw. 11. catheter.tw. 12. scalpel.tw. 13. surgery.tw. 14. surgical.tw. 15. operat*.tw. 16. interventional.tw. 17. device*.tw. 18. implant*.tw. 19. prosthe*.tw. 20. robotic*.tw. 21. exp Specialties, Surgical/ 22. exp Endoscopy/ 23. Radiology, Interventional/ 24. exp Surgical Procedures, Operative/ 25. exp "prostheses and implants"/ 26. or/1-25 |
| **AND** |
| **Reviews (filter search)** |
| (((systematic OR state-of-the-art OR scoping OR narrative OR literature OR umbrella) ADJ (review* OR overview* OR assessment*)) OR review OR "review* of reviews" OR ((systematic OR evidence) ADJ1 assess*) OR "research evidence" OR metasynthe* OR meta-synthe*).tw. OR exp Review Literature as Topic/ OR exp Review/ OR "systematic review"/ |
| **AND** |
| **Human studies (filter search)** |
| 1. humans/ 2. animals/ 3. 1 not 2 |

***TABLE S2 – Search strategy for opinion pieces***

| **Definition, perception, classification** |
| --- |
| 1. Glossar*.tw. 2. Typolog*.tw. 3. Categori*.tw. 4. Framework?.tw. 5. Taxonom*.tw. 6. Recommendation?.tw. 7. Codif*.tw. 8. Catalogu*.tw. 9. Terminolog*.tw. 10. Concept*.tw. 11. diffusion of innovation/ 12. ((defin* or classif* or type?) adj3 (modification? or change?)).tw. 13. (method? adj2 (describ*3 or classif*3 or report*3 or categor*)).tw. 14. or/1-12 |
| **AND** |
| **Modifications** |
| 1. Modification?.tw. 2. (Incremental adj3 innovation?).tw 3. (adjust* or iterat* or alternat* or adapt* or revis* or switch* or custom* or re-work* or tweak* or tinker* or refin* modif* or improv* or combin* or nuance* or evolv* or add* or var* or chang* or alter* or deviat* or develop* or differ* or similar) adj10 (surg* or operat* or intra-operative or peri-operative or procedure* or device?).tw 4. OR 1-3 |
| **AND** |
| **Invasive procedure** |
| 1. invasive.tw. 2. incision.tw. 3. cut.tw. 4. percutaneous.tw. 5. puncture.tw. 6. (natural adj1 orifice).tw. 7. endoscop*.tw. 8. colonoscop*.tw. 9. gastroscop*.tw. 10. laparoscop*.tw. 11. catheter.tw. 12. scalpel.tw. 13. surgery.tw. 14. surgical.tw. 15. operat*.tw. 16. interventional.tw. 17. device*.tw. 18. implant*.tw. 19. prosthe*.tw. 20. robotic*.tw. 21. exp Specialties, Surgical/ 22. exp Endoscopy/ 23. Radiology, Interventional/ 24. exp Surgical Procedures, Operative/ 25. exp "prostheses and implants"/ 26. or/1-25 |
| **AND** |
| **Opinion pieces (filter search)** |
| 1. letter/ 2. editorial/ 3. comment/ 4. news/ 5. newspapers as topic/ 6. Newspaper Article/ 7. Expression of Concern/ 8. Editorial Policies/ 9. Anecdotes as topic/ 10. Guidelines as Topic/ 11. Advisory Committees/ 12. (scientific adj communication).ti 13. Comment*.ti 14. opinion?.ti 15. letter?.ti 16. editorial?.ti 17. view?.ti 18. (expert adj (review? or recommendation?)).ti 19. (expert opinion or current opinion).jw 20. or/1-19 |
| **AND** |
| **Human studies (filter search)** |
| 1. humans/ 2. animals/ 3. 1 not 2 |

# **Data** Extraction

***TABLE S3 – Summary of data extraction proforma***

| 1. **Publication Details** | - 1. First author   2. Journal   3. Title   4. Year |
| --- | --- |
| **2. Conflict of Interest** | - 1. Does the article provide a COI statement? (record verbatim)   2. Did the study receive funding and/or sponsorship? (record verbatim) |
| 1. **Publication Type** | - 1. Did the authors identify the publication type?   2. Reviewer-identified publication type   3. Method of determination of publication type |
| 1. **Author details** | - 1. Record the stated type of organization the author(s) are affiliated to (private, public, higher education)   2. Did the authors represent any of the following areas of expertise (surgeon, academic, methodologist, other)?   3. Country of origin   4. E-mail address of corresponding author   5. Was there any further information about the author(s)? E.g. expertise, role, specialty |
| 1. **Content of Article** | - 1. What was the purpose of the article?   2. What was the general context of the article?   3. Were there other information relevant to surgical innovation? (e.g. surgical training) |
| 1. **Definitions** | - 1. Does the article provide a definition of modifications? (record verbatim) |
| 1. **Perceptions and/or views** | - 1. Does the article discuss author perceptions of modifications? (record verbatim) |
| 1. **Classification, Reporting** | - 1. Does the article describe how modifications may be classified or reported? (record verbatim)   2. Do the authors make reference to an existing framework? |
| 1. **Other** | - 1. Record any other relevant information about modifications (record verbatim) |

# **Data Table**

A list of all included articles and summary data can be found below. A complete file with all extracted data (including verbatim extracted sections) is available from the authors.

***Table S4: Summary of included articles***

| **First Author** | **Publication Year** | **Region** | **Publication Type** |
| --- | --- | --- | --- |
| Abel et al. (2) | 2006 | United States | Literature Review |
| Agich et al. (3) | 2001 | United States | Editorial |
| Avery et al. (4) | 2019 | Europe | Literature Review |
| Biffl et al. (5) | 2008 | United States | Guideline |
| Bilbro et al. (6) | 2020 | Europe | Guideline |
| Birchley et al. (7) | 2020 | Europe | Literature Review |
| Campbell et al. (8) | 2008 | Europe | Editorial |
| Coobs et al. (9) | 2015 | United States, Asia | Literature Review |
| Currie et al. (10) | 2015 | Europe | Systematic Review |
| Das et al. (11) | 2019 | United States, Canada | Commentary |
| Diener et al. (12) | 2011 | Europe | Literature Review |
| Dymond et al. (13) | 2011 | Europe | Literature Review |
| Ergina et al. (14) | 2010 | United States, Canada, Europe | Literature Review |
| Ezaldein et al. (15) | 2018 | United States, Europe | Database Review |
| Faris et al. (16) | 2017 | United States | Literature Review |
| Garber et al. (17) | 2010 | United States | Commentary |
| Hansson et al. (18) | 2019 | Europe | Systematic Review |
| Hirst et al. (19) | 2019 | Europe | Guideline |
| Hirst et al. (20) | 2013 | Europe | Editorial |
| Kesselheim et al. (21) | 2014 | United States | Editorial |
| Lau et al. (22) | 2017 | Asia | Literature Review |
| Mangir et al. (23) | 2019 | Europe | Literature Review |
| Margo et al. (24) | 2001 | United States | Literature Review |
| Mastroianni et al. (25) | 2006 | United States | Literature Review |
| McCulloch et al. (26) | 2018 | Europe, United States | Literature Review |
| McCulloch et al. (27) | 2009 | Europe, United Sates, Canada | Literature Review |
| McCulloch et al. (28) | 2008 | Europe | Commentary |
| McCulloch et al. (29) | 2002 | Europe, Asia | Opinion Piece |
| McKneally et al. (30) | 2003 | Canada | Guideline |
| McLeod et al. (31) | 1999 | Canada | Commentary |
| Morreim et al. (32) | 2006 | United States | Debate |
| Olaiya et al. (33) | 2020 | Canada | Database Review |
| Pennell et al. (34) | 2016 | United States, Canada, Europe | Guideline |
| Pollock et al. (35) | 2020 | United States | Commentary |
| Rathi et al. (36) | 2015 | United States | Database Review |
| Riskin et al. (37) | 2006 | United States | Literature Review |
| Rome et al. (38) | 2014 | United States | Database Review |
| Sedrakyan et al. (39) | 2010 | United States | Guideline |
| Senturk et al. (40) | 2018 | Europe | Literature Review |
| Sorenson et al. (41) | 2014 | Europe | Literature Review |
| Starnes et al. (42) | 2013 | United States | Commentary |
| Stefanidis et al. (43) | 2014 | United States | Guideline |
| Strasberg et al. (44) | 2003 | United States | Literature Review |
| Sussman et al. (45) | 2000 | United States | Literature Review |
| Thompson et al. (46) | 2015 | United States | Literature Review |
| Turman et al. (47) | 2009 | United States | Literature Review |
| Waninger et al. (48) | 2013 | United States | Literature Review |
| Youngerman et al. (49) | 2015 | Europe | Opinion Piece |
| Zheng et al. (50) | 2014 | United States | Opinion Piece |

# Classification systems, taxonomies, views on reporting

***TABLE S5 – Summary of classification systems, taxonomies or typologies for categorising or reporting modifications***

| **Framework** | **Description** | **Reference** |
| --- | --- | --- |
| **Classification framework for defining surgical interventions** | Article explored existing descriptions of surgical interventions in clinical trials, and developed a typology intended to support standardisation of surgical interventions in the context of surgical trials. The authors have developed categories for classification of: i) the whole surgical intervention, ii) component parts of the surgical intervention, and iii) individual steps. | Blencowe *et al.* 2013. (51) |
| **Medical Devices Agency (MDA) Alert** | Regulatory guidance that draws attention to the risks of ‘home-made devices’. Such devices might be considered to include: adaptations of non-medical products, modifications to existing medical devices, and off-label use of existing medical devices. | Campbell *et al.* 2008. (8) |
| **The IDEAL Framework** | A framework that describes the different stages of surgical innovation. Stage 2a and 2b refer to iterative modification of a surgical intervention. | McCulloch et al. 2009. (27) |
| **Plan, Do, Check, Act (PDCA) cycle** | A formal industrial continuous quality improvement technique that consists of implementation of change, observation of effect, subsequent comparison with prior results and a decision to adopt, reject or continue making further changes. | McCulloch et al. 2008. (28) |
| **Tracker studies** | A suggested practical approach to study design when evaluating innovative interventions with a likelihood of frequent, multiple developments and variants using carefully planned yet flexible protocols with sophisticated interim analyses. | McKneally et al. 2003. (30) |
| **Real-time learning** | An approach by which to analyse and draw lessons from innovative processes whilst they are under way (based on a study conducted on teams learning minimally invasive cardiac surgery). | Edmondson et al. 2001. (52) |
| **Template for Intervention Description and Replication (TIDieR) checklist** | A checklist to guide how interventions, such as new surgical techniques, should be reported in IDEAL studies to support transparency and completeness of reporting. | Hoffmann et al. 2014. (53) |
| **Premarket Approval (PMA) Supplement Types** | A regulatory approval process consisting of five different tracks based on types of changes to medical devices post-marketing (panel-track, 180-day, real-time, special and 30-day notice). Describes type of change and data required according to review track. | United States Food and Drug Administration. (54) |

# Themes related to perceptions of modifications

***TABLE S6 – Summary of themes for perceptions of modifications to surgical procedures and devices***

| **All Themes (n=13)** | **Themes relevant to procedures (n=9)** | **Themes relevant to devices (n=10)** | **Themes common to procedures and devices (n=6)** |
| --- | --- | --- | --- |
| Modifications as a process | X  (goal-directed, step-wise) | X  (continuous, dynamic) | X |
| Types of modifications | X | X | X |
| Magnitude of modifications | X  (frequency of modifications) | X | X |
| Drivers of modifications | X | X | X |
| Enablers of modifications | X | X | X |
| Relationship to formal research | X  (inappropriateness or lack of formal research) | X  (implications for clinical studies and evidence) | X |
| Phase or stage of surgical innovation | X |  |  |
| Failures and mistakes | X |  |  |
| Increased complexity | X |  |  |
| Transparency issues, particularly minor modifications |  | X |  |
| Collaboration and joint working with industry |  | X |  |
| Access to innovation |  | X |  |
| Relationship between learning curve and patient selection |  | X |  |

# Summary of common themes

***TABLE S7 – Common themes for procedures and devices from thematic analysis of data on perceptions of modifications***

| **Theme** | **Example from included articles** | **Reference** |
| --- | --- | --- |
| **Process** | “At these stages [of procedure development], progress consists of limited and iterative steps.”  “Device manufacturers may frequently have improvements or minor changes to approved devices” | Das *et al.* (11)  Zheng *et al.* (50) |
| **Types/understandings of modifications** | “The surgical innovator has historically been allowed to “tinker” with procedures, introducing modifications of varying degrees to the point that a procedure could arguably be called new.”  “manufacturers seek to introduce major new design changes” | Biffl *et al.* (5)  Kesselheim *et al.* (21) |
| **Magnitude** | “While for minor device or procedure modifications only familiarization of the surgeon may be necessary, for more substantial changes, a more elaborate introduction process should be followed that may include familiarization, cognitive training, hands-on practice, performance assessment, patient disclosure, proctoring, and local and national outcome monitoring with the new device or procedure.”  “…ranging from minor design modifications to more substantial changes, such as adding a new use indication.” | Stefanidis *et al.* (43)  Ezaldein *et al.* (15) |
| **Drivers** | “Challenges are discussed and mistakes identified. The procedure is modified as needed.”  “Device modifications may be motivated by a need to improve performance or clinical outcomes, or both, or by an opportunity to expand the applicability of a device to more advanced disease or a new patient population.” | Das *et al.* (11)  Waninger *et al.* (48) |
| **Enablers** | “Iterative change is also germane to the notion of ‘enabling technologies’—existing innovations whose dissemination allows further innovations to arise.”  “…clinical success with a device often suggests modifications that could broaden the applicability of the device” | Birchley *et al.* (7)  Waninger *et al.* (48) |
| **Relationship to formal research** | “…how iteration of innovation is conceived may affect the level of governance that the innovation receives....”  “…progressive device modifications over a particular time period have implications for comparative studies in that it may or may not be acceptable to pool patient-level data directly across pre- and post-modification time periods.” | Birchley *et al.* (7)  Sedrakyan *et al.* (39) |

# Draft conceptual frameworks

***FIGURE S1 – Conceptual framework for procedure modifications***

**
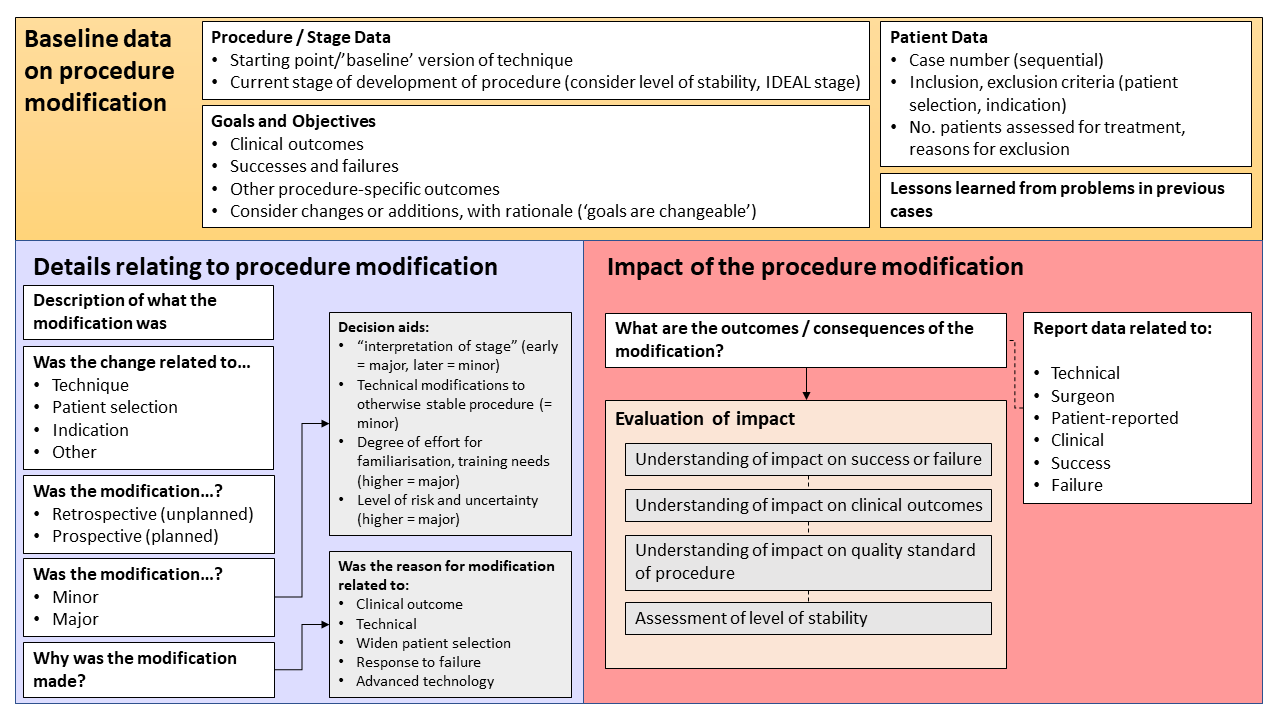
**

***FIGURE S2 – Conceptual framework for device modifications***

**
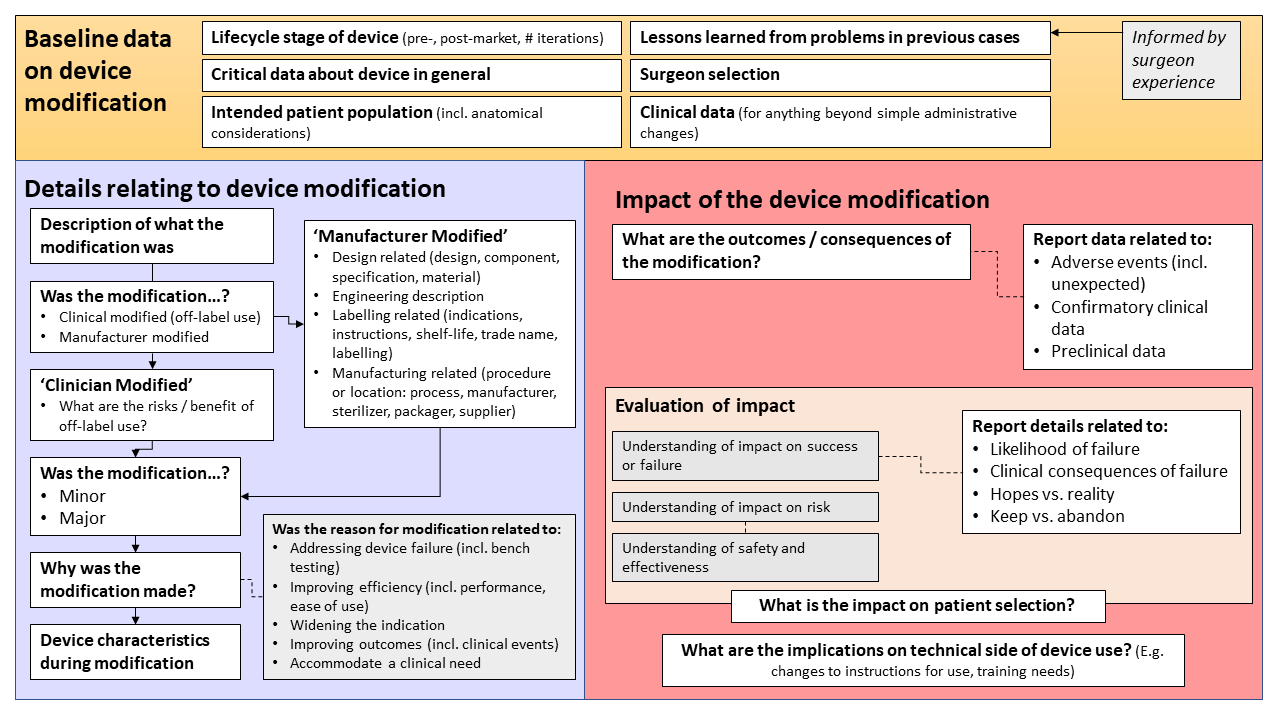
**

# Preferred Reporting Items for Systematic reviews and Meta-Analyses extension for Scoping Reviews (PRISMA-ScR) Checklist

**Appendix S1 – PRISMA-ScR Checklist**

| **SECTION** | **ITEM** | **PRISMA-ScR CHECKLIST ITEM** | **REPORTED ON PAGE #** |
| --- | --- | --- | --- |
| **TITLE** | | | |
| Title | 1 | Identify the report as a scoping review. | 1 |
| **ABSTRACT** | | | |
| Structured summary | 2 | Provide a structured summary that includes (as applicable): background, objectives, eligibility criteria, sources of evidence, charting methods, results, and conclusions that relate to the review questions and objectives. | 4 |
| **INTRODUCTION** | | | |
| Rationale | 3 | Describe the rationale for the review in the context of what is already known. Explain why the review questions/objectives lend themselves to a scoping review approach. | 6, Protocol Paper |
| Objectives | 4 | Provide an explicit statement of the questions and objectives being addressed with reference to their key elements (e.g., population or participants, concepts, and context) or other relevant key elements used to conceptualize the review questions and/or objectives. | 6 |
| **METHODS** | | | |
| Protocol and registration | 5 | Indicate whether a review protocol exists; state if and where it can be accessed (e.g., a Web address); and if available, provide registration information, including the registration number. | 6 |
| Eligibility criteria | 6 | Specify characteristics of the sources of evidence used as eligibility criteria (e.g., years considered, language, and publication status), and provide a rationale. | 7 |
| Information sources* | 7 | Describe all information sources in the search (e.g., databases with dates of coverage and contact with authors to identify additional sources), as well as the date the most recent search was executed. | 7 |
| Search | 8 | Present the full electronic search strategy for at least 1 database, including any limits used, such that it could be repeated. | Supplementary Material |
| Selection of sources of evidence† | 9 | State the process for selecting sources of evidence (i.e., screening and eligibility) included in the scoping review. | 7, 8 |
| Data charting process‡ | 10 | Describe the methods of charting data from the included sources of evidence (e.g., calibrated forms or forms that have been tested by the team before their use, and whether data charting was done independently or in duplicate) and any processes for obtaining and confirming data from investigators. | 8, Supplementary Material |
| Data items | 11 | List and define all variables for which data were sought and any assumptions and simplifications made. | 8, Supplementary Material |
| Critical appraisal of individual sources of evidence§ | 12 | If done, provide a rationale for conducting a critical appraisal of included sources of evidence; describe the methods used and how this information was used in any data synthesis (if appropriate). | N/A |
| Synthesis of results | 13 | Describe the methods of handling and summarizing the data that were charted. | 8,9 |
| **RESULTS** | | | |
| Selection of sources of evidence | 14 | Give numbers of sources of evidence screened, assessed for eligibility, and included in the review, with reasons for exclusions at each stage, ideally using a flow diagram. | 9,10 |
| Characteristics of sources of evidence | 15 | For each source of evidence, present characteristics for which data were charted and provide the citations. | 10,11 |
| Critical appraisal within sources of evidence | 16 | If done, present data on critical appraisal of included sources of evidence (see item 12). | N/A |
| Results of individual sources of evidence | 17 | For each included source of evidence, present the relevant data that were charted that relate to the review questions and objectives. | Supplementary Material |
| Synthesis of results | 18 | Summarize and/or present the charting results as they relate to the review questions and objectives. | 12,13,14,15,16 |
| **DISCUSSION** | | | |
| Summary of evidence | 19 | Summarize the main results (including an overview of concepts, themes, and types of evidence available), link to the review questions and objectives, and consider the relevance to key groups. | 16, 17 |
| Limitations | 20 | Discuss the limitations of the scoping review process. | 18, 19 |
| Conclusions | 21 | Provide a general interpretation of the results with respect to the review questions and objectives, as well as potential implications and/or next steps. | 20 |
| **FUNDING** | | | |
| Funding | 22 | Describe sources of funding for the included sources of evidence, as well as sources of funding for the scoping review. Describe the role of the funders of the scoping review. | 2 |

JBI = Joanna Briggs Institute; PRISMA-ScR = Preferred Reporting Items for Systematic reviews and Meta-Analyses extension for Scoping Reviews.

* Where *sources of evidence* (see second footnote) are compiled from, such as bibliographic databases, social media platforms, and Web sites.

† A more inclusive/heterogeneous term used to account for the different types of evidence or data sources (e.g., quantitative and/or qualitative research, expert opinion, and policy documents) that may be eligible in a scoping review as opposed to only studies. This is not to be confused with *information sources* (see first footnote).

‡ The frameworks by Arksey and O’Malley (6) and Levac and colleagues (7) and the JBI guidance (4, 5) refer to the process of data extraction in a scoping review as data charting*.*

§ The process of systematically examining research evidence to assess its validity, results, and relevance before using it to inform a decision. This term is used for items 12 and 19 instead of "risk of bias" (which is more applicable to systematic reviews of interventions) to include and acknowledge the various sources of evidence that may be used in a scoping review (e.g., quantitative and/or qualitative research, expert opinion, and policy document).

*From:* Tricco AC, Lillie E, Zarin W, O'Brien KK, Colquhoun H, Levac D, et al. PRISMA Extension for Scoping Reviews (PRISMAScR): Checklist and Explanation. Ann Intern Med. 2018;169:467–473. [doi: 10.7326/M18-0850](http://annals.org/aim/fullarticle/2700389/prisma-extension-scoping-reviews-prisma-scr-checklist-explanation).

# References

1. Hoffmann C, Hossaini S, Cousins S, Blencowe N, McNair AGK, Blazeby JM, et al. Reporting Modifications in Surgical Innovation: A Systematic Scoping Review Protocol. Int J Surg Protoc. 2021;25(1):250-6.

2. Abel DB, Dehdashtian MM, Rodger ST, Smith AC, Smith LJ, Waninger MS. Evolution and future of preclinical testing for endovascular grafts. J Endovasc Ther. 2006;13(5):649-59.

3. Agich GJ. Ethics and innovation in medicine. J Med Ethics. 2001;27(5):295-6.

4. Avery K, Blazeby J, Wilson N, Macefield R, Cousins S, Main B, et al. Development of reporting guidance and core outcome sets for seamless, standardised evaluation of innovative surgical procedures and devices: a study protocol for content generation and a Delphi consensus process (COHESIVE study). BMJ Open. 2019;9(9):e029574.

5. Biffl WL, Spain DA, Reitsma AM, Minter RM, Upperman J, Wilson M, et al. Responsible development and application of surgical innovations: a position statement of the Society of University Surgeons. J Am Coll Surg. 2008;206(6):1204-9.

6. Bilbro NA, Hirst A, Paez A, Vasey B, Pufulete M, Sedrakyan A, et al. The IDEAL Reporting Guidelines: A Delphi Consensus Statement Stage specific recommendations for reporting the evaluation of surgical innovation. Ann Surg. 2020.

7. Birchley G, Ives J, Huxtable R, Blazeby J. Conceptualising Surgical Innovation: An Eliminativist Proposal. Health Care Anal. 2020;28(1):73-97.

8. Campbell B. Home-made, adapted and modified devices in surgical practice. Ann R Coll Surg Engl. 2008;90(3):251-2.

9. Coobs BR, Xiong A, Clohisy JC. Contemporary Concepts in the Young Adult Hip Patient: Periacetabular Osteotomy for Hip Dysplasia. J Arthroplasty. 2015;30(7):1105-8.

10. Currie A, Brigic A, Blencowe NS, Potter S, Faiz OD, Kennedy RH, et al. Systematic review of surgical innovation reporting in laparoendoscopic colonic polyp resection. Br J Surg. 2015;102(2):e108-16.

11. Das S, McKneally M. The Surgeon-in-Chief Should Oversee Innovative Surgical Practice. Am J Bioeth. 2019;19(6):34-6.

12. Diener MK, Simon T, Büchler MW, Seiler CM. Surgical evaluation and knowledge transfer--methods of clinical research in surgery. Langenbecks Arch Surg. 2012;397(8):1193-9.

13. Dymond E, Long A, McCarthy A, Drake MJ. Developing a new treatment device: how to get an idea to the marketplace. Neurourol Urodyn. 2012;31(4):429-36.

14. Ergina PL, Cook JA, Blazeby JM, Boutron I, Clavien PA, Reeves BC, et al. Challenges in evaluating surgical innovation. Lancet. 2009;374(9695):1097-104.

15. Ezaldein HH, Scott JF, Yin ES, Ventura A, DeRuyter NP, Leffell DJ. Transparency and Dermatologic Device Approval by the US Food and Drug Administration. JAMA Dermatol. 2018;154(3):273-80.

16. Faris O, Shuren J. An FDA Viewpoint on Unique Considerations for Medical-Device Clinical Trials. N Engl J Med. 2017;376(14):1350-7.

17. Garber AM. Modernizing device regulation. N Engl J Med. 2010;362(13):1161-3.

18. Hansson E, Jepsen C, Hallberg H. Breast reconstruction with a dermal sling: a systematic review of surgical modifications. J Plast Surg Hand Surg. 2019;53(1):1-13.

19. Hirst A, Philippou Y, Blazeby J, Campbell B, Campbell M, Feinberg J, et al. No Surgical Innovation Without Evaluation: Evolution and Further Development of the IDEAL Framework and Recommendations. Ann Surg. 2019;269(2):211-20.

20. Hirst A, Agha RA, Rosin D, McCulloch P. How can we improve surgical research and innovation?: the IDEAL framework for action. Int J Surg. 2013;11(10):1038-42.

21. Kesselheim AS, Rajan PV. Regulating incremental innovation in medical devices. BMJ. 2014;349:g5303.

22. Lau WY, Lai EC. Modifications of ALPPS - from complex to more complex or from complex to less complex operations. Hepatobiliary Pancreat Dis Int. 2017;16(4):346-52.

23. Mangir N, Roman S, MacNeil S. The changing regulatory landscape for biomedical implants and its relationship to withdrawal of some vaginal mesh products. Curr Opin Urol. 2019;29(4):414-8.

24. Margo CE. When is surgery research? Towards an operational definition of human research. J Med Ethics. 2001;27(1):40-3.

25. Mastroianni AC. Liability, regulation and policy in surgical innovation: the cutting edge of research and therapy. Health Matrix Clevel. 2006;16(2):351-442.

26. McCulloch P, Feinberg J, Philippou Y, Kolias A, Kehoe S, Lancaster G, et al. Progress in clinical research in surgery and IDEAL. Lancet. 2018;392(10141):88-94.

27. McCulloch P, Altman DG, Campbell WB, Flum DR, Glasziou P, Marshall JC, et al. No surgical innovation without evaluation: the IDEAL recommendations. Lancet. 2009;374(9695):1105-12.

28. McCulloch P. Developing appropriate methodology for the study of surgical techniques. J R Soc Med. 2009;102(2):51-5.

29. McCulloch P, Taylor I, Sasako M, Lovett B, Griffin D. Randomised trials in surgery: problems and possible solutions. BMJ. 2002;324(7351):1448-51.

30. McKneally MF, Daar AS. Introducing new technologies: protecting subjects of surgical innovation and research. World J Surg. 2003;27(8):930-4; discussion 4-5.

31. McLeod RS. Issues in surgical randomized controlled trials. World J Surg. 1999;23(12):1210-4.

32. Morreim H, Mack MJ, Sade RM. Surgical innovation: too risky to remain unregulated? Ann Thorac Surg. 2006;82(6):1957-65.

33. Olaiya OR, Oyesile D, Stone N, Mbuagbaw L, McRae MH. Postmarket Modifications of High-risk Plastic Surgery Devices. Plast Reconstr Surg Glob Open. 2020;8(2):e2621.

34. Pennell CP, Hirst AD, Campbell WB, Sood A, Agha RA, Barkun JS, et al. Practical guide to the Idea, Development and Exploration stages of the IDEAL Framework and Recommendations. Br J Surg. 2016;103(5):607-15.

35. Pollock TA. Commentary on: Reduced Seroma Risk in Drainless Abdominoplasty Using Running Barbed Sutures: A 10-Year, Multicenter Retrospective Analysis. Aesthet Surg J. 2020;40(5):538-40.

36. Rathi VK, Ross JS, Samuel AM, Mehra S. Postmarket Modifications of High-Risk Therapeutic Devices in Otolaryngology Cleared by the US Food and Drug Administration. Otolaryngol Head Neck Surg. 2015;153(3):400-8.

37. Riskin DJ, Longaker MT, Gertner M, Krummel TM. Innovation in surgery: a historical perspective. Ann Surg. 2006;244(5):686-93.

38. Rome BN, Kramer DB, Kesselheim AS. FDA approval of cardiac implantable electronic devices via original and supplement premarket approval pathways, 1979-2012. JAMA. 2014;311(4):385-91.

39. Sedrakyan A, Marinac-Dabic D, Normand SL, Mushlin A, Gross T. A framework for evidence evaluation and methodological issues in implantable device studies. Med Care. 2010;48(6 Suppl):S121-8.

40. Şentürk MF, Yazıcı T, Gülşen U. Techniques and modifications for TMJ arthrocentesis: A literature review. Cranio. 2018;36(5):332-40.

41. Sorenson C, Drummond M. Improving medical device regulation: the United States and Europe in perspective. Milbank Q. 2014;92(1):114-50.

42. Starnes BW. A surgeon's perspective regarding the regulatory, compliance, and legal issues involved with physician-modified devices. J Vasc Surg. 2013;57(3):829-31.

43. Stefanidis D, Fanelli RD, Price R, Richardson W, Committee SG. SAGES guidelines for the introduction of new technology and techniques. Surg Endosc. 2014;28(8):2257-71.

44. Strasberg SM, Ludbrook PA. Who oversees innovative practice? Is there a structure that meets the monitoring needs of new techniques? J Am Coll Surg. 2003;196(6):938-48.

45. Sussman MD. Ethical requirements that must be met before the introduction of new procedures. Clin Orthop Relat Res. 2000(378):15-22.

46. Thompson CH, Zoratti MJ, Langhals NB, Purcell EK. Regenerative Electrode Interfaces for Neural Prostheses. Tissue Eng Part B Rev. 2016;22(2):125-35.

47. Turman KA, Diduch DR, Miller MD. All-inside meniscal repair. Sports Health. 2009;1(5):438-44.

48. Waninger MS, Whirley RG, Smith LJ, Wolf BS. Manufacturer evaluations of endograft modifications. J Vasc Surg. 2013;57(3):826-8.

49. Youngerman BE, McKhann GM, 2nd. Innovation in surgery and evidence development: can we have both at once? Virtual Mentor. 2015;17(1):41-8.

50. Zheng SY, Redberg RF. Premarket approval supplement pathway: do we know what we are getting? Ann Intern Med. 2014;160(11):798-9.

51. Blencowe N, Boddy A, Harris A, Hanna T, Whiting P, Cook J, et al. Accounting for intervention complexity in rcts in surgery: new approaches for intervention definition and methods for monitoring fidelity. Trials. 2013;14(1):O86.

52. Edmondson A, Bohmer R, Pisano G. Speeding Up Team Learning. Harvard Business Review. 2001;79(9):125-32.

53. Hoffmann TC, Glasziou PP, Boutron I, Milne R, Perera R, Moher D, et al. Better reporting of interventions: template for intervention description and replication (TIDieR) checklist and guide. BMJ. 2014;348:g1687.

54. Administration USFaD. PMA Supplements and Amendments. In: Devices M, editor. 2019.
